# Ion transport and limited currents in supporting electrolytes and ionic liquids

**DOI:** 10.1038/s41598-022-10183-2

**Published:** 2022-04-13

**Authors:** Maximilian Schalenbach, Yasin Emre Durmus, Hermann Tempel, Hans Kungl, Rüdiger-A. Eichel

**Affiliations:** Fundamental Electrochemistry (IEK‑9), Institute of Energy and Climate Research, Forschungszentrum Jülich GmbH, 52425 Jülich, Germany

**Keywords:** Batteries, Chemistry, Electrochemistry, Inorganic chemistry, Physical chemistry, Theoretical chemistry

## Abstract

Supporting electrolytes contain inert dissolved salts to increase the conductivity, to change microenvironments near the electrodes and to assist in electrochemical reactions. This combined experimental and computational study examines the impact of supporting salts on the ion transport and related limited currents in electrochemical cells. A physical model that describes the multi-ion transport in liquid electrolytes and the resulting concentration gradients is presented. This model and its parameterization are evaluated by the measured limited current of the copper deposition in a CuSO_4_ electrolyte under a gradually increasing amount of Na_2_SO_4_ that acts as a supporting salt. A computational sensibility analysis of the transport model reveals that the shared conductance between the ions lowers the limited currents with larger supporting salt concentrations. When the supporting salt supplies most of the conductance, the electric-field-driven transport of the electrochemically active ions becomes negligible so that the limited current drops to the diffusion-limited current that is described by Fick’s first law. The transition from diluted supporting electrolyte to the case of ionic liquids is elucidated with the transport model, highlighting the different physical transport mechanisms in a non-conducting (polar) and a conducting (ionic) solvent.

## Introduction

Supporting electrolytes are solvent-based liquids that contain inert salt additives (defined as supporting salts) which are not electrochemically converted. These additive salts can increase the conductivity of the electrolyte and thereby improve the efficiency of electrochemical processes^1–3^. For example, in industrial copper refining and winning the conductivity of aqueous CuSO_4_ baths is increased by H_2_SO_4_ addition^4,5^. Electrolytes with at least three different sorts of ions also emerge when reactive ion types are dissolved with inert ions in for instance aqueous salt solutions^6–8^, molten salts^9–11^, deep eutectic solvents^12,13^, or ionic liquids^14–17^.

For binary (one type of cation and one type of anion) solvent-based electrolytes the transport of ions was experimentally characterized and theoretically described in various works^18–20^. In these systems, the electrolyte is thinning out at the electrode at which dissolved ions are electrochemically converted, whereas the ions accumulate at the electrode which introduces ions into the electrolyte. Thus, electrochemical reactions cause concentration gradients in liquid electrolytes^21–23^ that ultimately limit the current that electric-field and diffusion-driven ion transport can carry without locally depleting the concentration of the converted ion types^24^. The limited currents and concentration gradients in multi-ion systems with its relation to the microenvironment at the electrode (such as pH^25,26^) display an important design aspect for electrochemical devices for batteries^21–23^, the separation of ions^27,28^, CO_2_ reduction^29,30^, electrowinning^31,32^, electroplating^33–35^ and so forth.

In binary electrolytic solutions, the electrolyte parameterization is described by the transfer numbers (that describe the different contributions of the anions and cations to the conduction), the mutual diffusion coefficient (pairwise diffusion of anions and cations under the boundary condition of electro-neutrality in the solution) and the molar conductivity. These properties depend on the concentration, for which the electrochemically driven concentration gradients require a spatially resolved parameterization for a precise transport modeling^24^. Numerical time domain modeling of the electrochemical transport differential equations has proven its reliability to represent the concentration dependence of the electrolyte parameterization, while it also can resolve changing boundary conditions such as varying currents^24^.

The ion transport in supporting electrolytes was modeled in previous studies with analytical^36–38^ and numerical^39–41^ approaches to solve the electrochemical transport equations. From these studies, only Awakura et al.^37^ compared model results with experimental data, which is however crucial to understand and describe the complexity of the ion–ion interactions in these systems that cannot be precisely described by ab-initio models. All of these studies were conducted with a constant set of electrolyte parameters that do not take into account the concentration dependence and ion–ion interactions. Most of these studies considered an infinite distance of anode and cathode^37–39,41^, which simplifies the mathematical description and analytical solution of the transport equations. This scenario however does not lead to a steady state with a constant limited current^24^. A detailed understanding of the mechanisms that influence limited current in supported electrolytes, its correlation to the electrolyte properties and the parameterization of the interaction of different sort of ions is not yet reported.

The aim of this study is to describe the ion transport in supporting electrolytes with a physical model of the electrochemical transport equations which enables a concentration-dependent parameterization and the description of steady limited currents. The modeled limited currents are compared under different parameterization scenarios to measured limited currents of mixed CuSO_4_ and Na_2_SO_4_ electrolytes. To understand the physicochemical mechanisms of the ion transport in supported electrolytes and its influence on the limited current in detail, a computationally sensibility analysis of the parameterization is conducted. Moreover, the computer model is adjusted to multi-ion systems in molten salts and ionic liquids, showing similarities and differences of the ion transport in comparison to the solvent-based electrolytes. The source codes of the computational model are provided in the supporting information, so that the community effortlessly can reproduce the presented results and thereon further develop the presented model.

## Methods

### Experimental

In this study, the copper deposition and dissolution of plane and polished copper electrodes in mixed CuSO_4_ and Na_2_SO_4_ electrolytes serve as an exemplary system of supporting electrolytes to examine limited currents. Hereto, the two polished copper plates are separated by a stamped fluoroelastomer flat sealing with an inner diameter of 14 mm and a thickness of 500 µm (Reichelt Chemietechnik). To assemble the cell, the sealing was laid on one electrode and the copper surface was wetted with approximately 1 ml of electrolyte. The surface of the other electrode was also wetted with the electrolyte and then pressed onto the electrode with the sealing. Thereby, the excess electrolyte floated out of the cell assembly while the sealing between the electrodes was completely filled with the electrolyte. The anode was placed at the bottom to avoid macroscopic density differences that are compensated by gravitational shear forces^24^. An additional cell with copper electrodes and an electrode distance of 20 mm was used to characterize the conductivity of Na_2_SO_4_ solutions. Hereto, the copper electrodes were pressed with flat sealings (same type as above) on a polypropylene body. With 4-wire potentiodynamic alternating current impedance measurements, the electrolyte resistance was determined^42^.

### Model

A detailed model on the ion transport in binary electrolytes has been presented previously^24^, which included an experimental evaluation and a concentration-dependent parameterization of the molar conductivity, mutual diffusion coefficient and ion transfer numbers. This transport model describes the electric field and diffusion driven motion of dissolved ions in liquid electrolytes and is in the following adapted to an electrolyte with three ion types. This multi-ion system is in the following parameterized on the basis of reported experimental conductivities, diffusion coefficients and transfer numbers on the binary electrolyte systems of CuSO_4_ and Na_2_SO_4_, respectively. The source codes of this approach are supplied in the supporting information.

**Figure 1:**
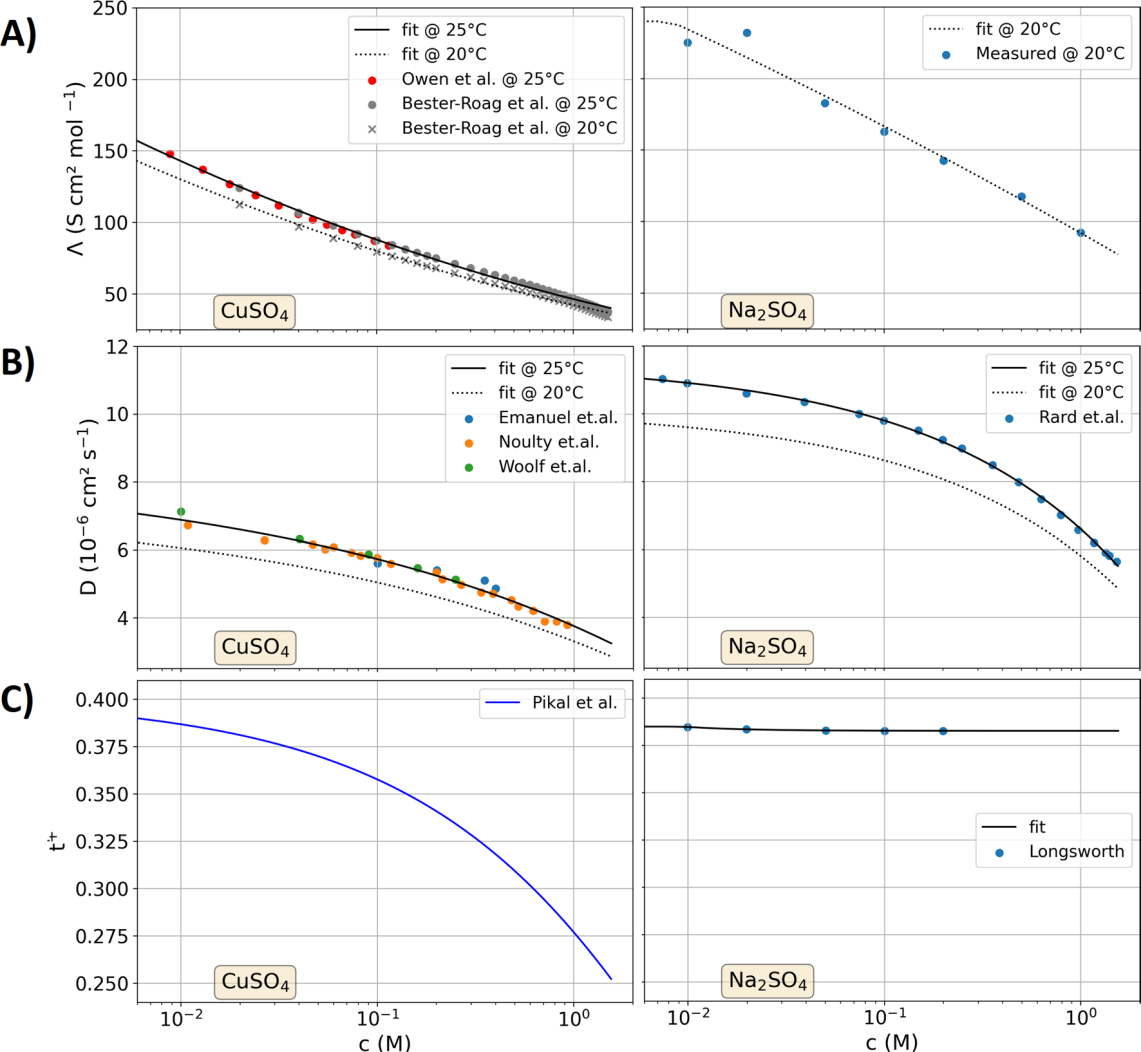
Parameterization of CuSO_4_ (left) and Na_2_SO_4_ (right) binary electrolytes as a function of the concentration. (**A**) Molar conductivity. (**B**) Diffusion coefficient. (**C**) Transfer number.

Figure 1 summarizes the data on molar conductivity, diffusion coefficient and cation transfer number of aqueous CuSO_4_ and Na_2_SO_4_ electrolyte solutions, respectively, referring to the data reported by Owen et al.^43^, Bester-Roag et al.^44^, Emanuel et al.^45^, Noulty et al.^46^, Woolf et al.^47^, Rard et al.^48^, Pikal et al.^49^ and Longsworth et al.^50^. The molar conductivity and diffusion coefficients decrease towards higher electrolyte concentrations, which is a direct result of the ion–ion interaction that is described by the Debye-Hückel theory^51,52^. Most of the literature reported values at the standard temperature of 25 °C, whereas the measurements in this study were performed at 20 °C. The effect of temperature on the molar conductivity and diffusion coefficient is discussed elsewhere in detail^24^. The conductivity and the transfer coefficient of the CuSO_4_ electrolyte show a more distinct concentration dependence than those of the Na_2_SO_4_ electrolyte. The dissolution of CuSO_4_ acidifies the aqueous solvent, however, the concentration of protons is more than 1000 smaller than the concentration of dissolved copper for which the impact on protons on the transport model is negligible^24^. The trends of the concentration dependence of the electrolyte properties can be described on the basis of the Debye–Hückel theory, whereas the complexity of the aqueous solutions do not allow a precise theoretical prediction of ion–ion interactions and their impact on conductivities and diffusion coefficients^53,54^. Thus, the experimental data is described by analytical non-physical equations which adequately describe the concentration dependence of the literature data in Fig. 1 as discussed in the supporting information in detail.

The transfer number ${t}^{i}$ of the ion type $i$ is defined by the current ${I}^{i}$ that it carries in relation to the total current ${I}_{\text{tot}}$:

1 $${t}^{i}= \frac{{I}^{i}}{{I}_{\text{tot}}}.$$

Using Ohm’s law, this equation can also be interpreted as a ratio of conductivities $\kappa$:

2 $${t}^{i}= \frac{{\kappa }^{i}}{{\kappa }_{\text{tot}}}.$$

To apply this definition to multi-ion systems, a more precise notation is introduced in the following. In Na_2_SO_4_, the anion (${\text{SO}}_{4}^{2-}$) has a valence of two whereas the cation (${\text{Na}}^{+}$) has a valence of one. By using the lowest common denominator of the valences, equal charges and equal concentrations of the definitions of $C=2{\text{Na}}^{+}$ and $A={\text{SO}}_{4}^{2-}$ result. The molar conductivity $\Lambda$ of an binary electrolyte that is comprised by type A and C is here defined as ${\Lambda }^{\text{AC}}$ (units of $\text{S c}\, {{\text{m}}^{2}} \,\text{ mol}^{-1}$). As shown in Fig. 1, the transfer number and the molar conductivity have an intrinsic concentration dependence for which both are a function of the concentration ${c}^{\text{AC}}$. The conductivity of the binary electrolyte ${\kappa }^{\text{AC}}$ equals the product of concentration and molar conductivity:

3 $${\kappa }^{\text{AC}}={c}^{\text{AC}}\times {\Lambda }^{\text{AC}}.$$

With the molar conductivity and the ion transport number, the conductivity ${\upkappa }^{\text{A}}$ of the anion $A$ as a function of the concentration can be calculated as

4 $${\upkappa }^{\text{A}}={c}^{\text{AC}}\times {t}^{\text{A}} \times {\Lambda }^{\text{AC}}.$$

In the considered multi-ion system of CuSO_4_ with Na_2_SO_4_ addition, the conductivity of the sodium and copper ions are determined similarly to the binary solutions

5 $${\upkappa }^{{\text{Cu}}^{2+}}={t}^{{\text{Cu}}^{2+}} {c}^{{\text{CuSO}}_{4}} {\widehat{\Lambda }}^{{\text{CuSO}}_{4}},$$

6 $${\upkappa }^{2{\text{Na}}^{+}}={t}^{2{\text{Na}}^{+}}{c}^{{\text{Na}}_{2}{\text{SO}}_{4}} {\widehat{\Lambda }}^{{\text{Na}}_{2}{\text{SO}}_{4}} ,$$

where $\widehat{\Lambda }$ includes the influence by the interactions between the different ion types on the molar conductivity. In the case of the sulfate anion, the situation becomes more complex, as it is involved in the conduction of both salts. Its conductivity is here calculated as the sum of its contributions to the conductivity of both dissolved salts:

7 $${\upkappa }^{{\text{SO}}_{4}^{2-}}=\left[1-{t}^{{\text{Cu}}^{2+}}\right] {c}^{{\text{CuSO}}_{4}}{ \widehat{\Lambda }}^{{\text{CuSO}}_{4}} + \left[1-{t}^{2{\text{Na}}^{+}}\right] {c}^{{\text{Na}}_{2}{\text{SO}}_{4}}{\widehat{\Lambda }}^{{\text{Na}}_{2}{\text{SO}}_{4}}.$$

In the results part, different approaches to estimate the influence of the ion interactions on the molar conductivity are presented, which are based on the data in Fig. 1. The total conductivity ${\kappa }_{\text{tot}}$ in the multi-ion system equals the sum of the conductivities of the individual types of ions. Accordingly, the conductivity ratio (Eq. (2)) can be determined for multi-ion systems, which expressed the share of the different ion types to the overall current.

The diffusion process of type A and C is always pairwise, as otherwise the electroneutrality is violated. Thus, the mutual diffusion coefficients $D$ are used to calculate those of the cations:

8 $${D}^{{\text{Cu}}^{2+}}={D}^{{\text{CuSO}}_{4}} ,$$

9 $${D}^{2{\text{Na}}^{+}}={D}^{{\text{Na}}_{2}{\text{SO}}_{4}}.$$

For the sulfate ions, the weighted arithmetic mean is used to calculate the diffusion coefficient, as their diffusion also depends on the mobility of the cations to which they are paired:

10 $${D}^{{\text{SO}}_{4}^{ 2-}}=\frac{{{c}^{{\text{CuSO}}_{4}} D}^{{\text{CuSO}}_{4}}+{c}^{{\text{Na}}_{2}{\text{SO}}_{4}} {D}^{{\text{Na}}_{2}{\text{SO}}_{4}}}{{c}^{{\text{CuSO}}_{4}}+{c}^{{\text{Na}}_{2}{\text{SO}}_{4}} }.$$

The differential equations of the transport model are discussed in the supporting information in detail, including their boundary conditions at the electrodes and their implementation in the numerical simulation framework.

## Results and discussion

First, the experimental and model data on the ionic transport of mixed CuSO_4_ and Na_2_SO_4_ electrolytes is presented. Second, the influence of the electrolyte parameters on limited currents is examined using the transport model. Moreover, the computer model is used to describe the transition from diluted polar solvents to ionic liquids, highlighting similarities and differences of both systems.

### Measured and modeled data on the CuSO_4_–Na_2_SO_4_ electrolytes

**Figure 2:**
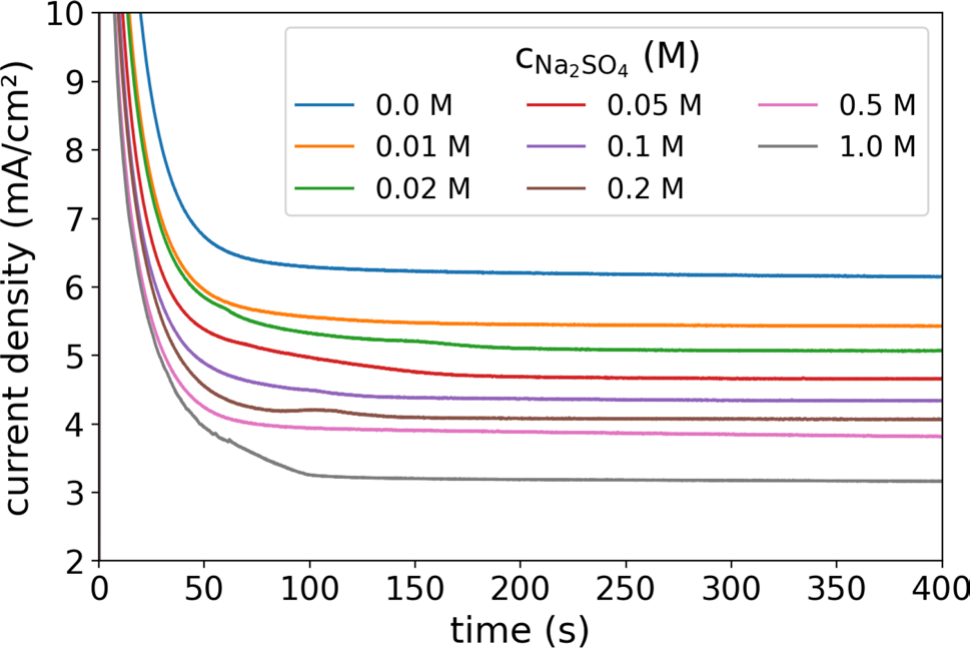
Measured current densities at 0.3 V between two copper electrodes (distance of 500 µm) with a CuSO_4_ concentration of 0.1 M and a variation of the Na_2_SO_4_ concentration from 0 to 1 M.

Figure 2 shows the measured current densities of a 0.1 M CuSO_4_ solution with a variation of Na_2_SO_4_ addition from 0 to 1 M as a function of time. A voltage of 0.3 V was applied between the electrodes that were 500 µm apart from each other (see “Experimental” section). After initial double layer charging and surface reduction/oxidation the current can be mainly attributed to the copper deposition and dissolution. This electrochemical current decays over time as the cathodic copper ion concentration depletes (detailed discussion below). After approximately 200 s a steady current is reached for all different concentrations.

**Figure 3:**
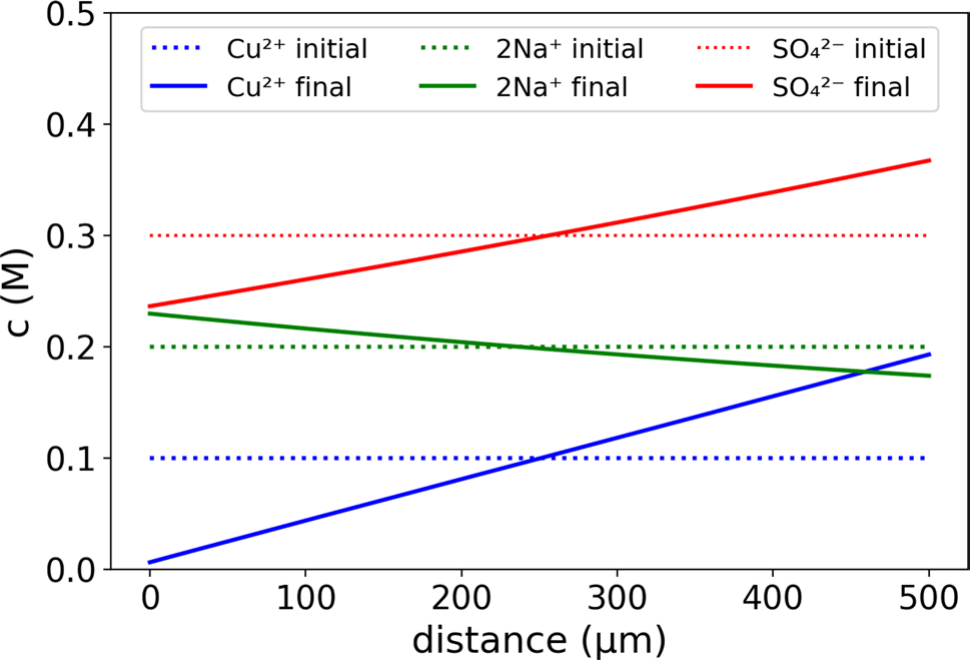
Modeled concentration gradients (using the parameterization case iv as defined in the text below) at the limited current for an electrode distance of 500 µm, a CuSO_4_ concentration of 0.1 M, and a Na_2_SO_4_ concentration of 0.2 M. The cathode is located at the left side (distance = 0) and the anode at the right side (distance = 500 µm).

To calculate the limited currents with the computational transport model, the simulation is started with a current density of 15 mA/cm^2^, resembling the experimental approach by beginning with a larger value than the expected limited current density. When the concentration of copper ions at the cathode depletes to 1/15 of the initial concentration of 0.1 M, the current is decreased. After this initial reduction, the current is continuously adjusted so that the cathodic copper ion concentration is ranging between 1/15 and 1/12 of the initial concentration. Figure 3 shows an example of the modeled concentration gradients between the electrodes for a total CuSO_4_ concentration of 0.1 M and a Na_2_SO_4_ concentration of 0.2 M. In the initial state, the concentration of the ions is evenly distributed in the electrolytic solution. In the final state, a steady limited current is reached, in which the concentration gradients do not change as a function of time and in which the copper ion concentration at the cathode is depleted. The total concentrations of the ions in the electrolyte do not change, as the inert ions are not converted at the electrodes and as the same amount of copper is deposited and dissolved. Moreover, the sum of the charges of the cations and anions is locally and globally zero, fulfilling the electroneutrality of the electrolyte (see detailed discussion in reference^24^).

In contrast to the copper ion starvation at the cathode and its accumulation at the anode, the sodium ion concentration gradient is converse, with an enlarged amount at the cathode and a reduced amount at the anode. The sodium ions are transported by the electric field in the electrolyte to the cathode where they cannot be converted. In the steady state, the sodium ion current by the electric field towards the cathode is balanced by diffusion into the opposite transition, leading to no net sodium ion transport. As the sulfate ions are transported by the electric field towards the anode, the amount of sulfate ions is decreased at the cathode and increased at the anode. As the sulfate ions are the only anions in this system, their amount reflects the total amount of dissolved ions with respect to the charge. Similar to the sodium ions, in the steady state the net sulfate ion transport equals zero so that the copper ions as the only electrochemical active ion type constitute the total net current. The electric-field and diffusion-driven ion transport of the copper ions point in the same direction from the anode to the cathode.

**Figure 4:**
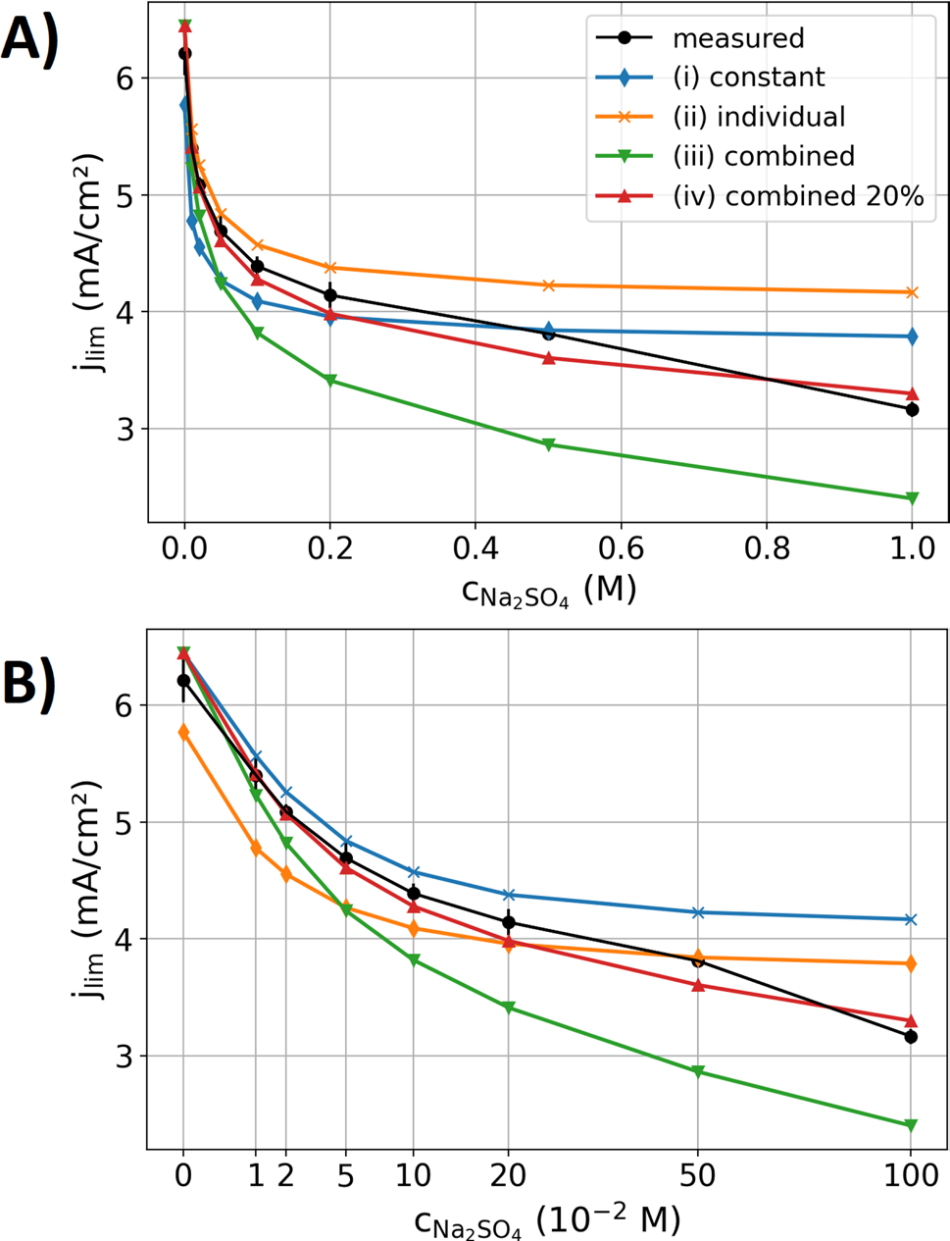
Measured (black lines with scatter and error bars) and modeled (colored lines) limited currents densities for a CuSO_4_ concentration of 0.1 M under a variation of the Na_2_SO_4_ concentration. The four different parameterization scenarios for the model are discussed in the text. The measured data is recorded with a voltage of 0.3 V between the electrodes. (**A**) Linear increment of the x-axis. (**B**) Square root spacing of the x-axis to more clearly resolve the limited current densities at small concentrations.

From the measurements graphed in Fig. 2, the limited current was determined by the mean current between 300 and 400 s after the experiments started. In addition to the data graphed in Fig. 2, two repetition measurements were conducted. Figure 4 shows the mean of these measurements with the standard variation as the statistic error of the experiments. Moreover, Fig. 4 shows the modeled data, which is considered for four different approaches of the parameterization, which all show similar trends as the experiment with decreasing limited currents towards higher Na_2_SO_4_ concentrations. The four different parameterizations are characterized by the following features:

(i) The first case is modeled with a constant parameterization, resembling previously reported models in the literature^36–41^. The values of Fig. 1 at 0.1 M concentration of CuSO_4_ and Na_2_SO_4_ serve as model input parameters. When the concentration of copper ions near the cathode is thinning out, their local conductivity, diffusion coefficient and transfer number increase with reference to Fig. 1 which increases the limited current in pure CuSO_4_ electrolytes^24^. By neglecting these concentration dependencies, the current is up to a Na_2_SO_4_ concentration of 0.5 M underestimated (similar to that in binary solutions^24^), as the locally higher diffusivity and ion transport number (see Fig. 1) of the copper ions at the depleting cathodic concentration is not taken into account. At higher Na_2_SO_4_ concentrations, the decrease of the conductivity, diffusion coefficients and cation transfer number becomes significant so that the model with the constant parameterization overestimates the limited current.
(ii) In the second case, the model is parameterized with the individual concentration dependencies of CuSO_4_ and Na_2_SO_4_ respectively, without taking their interaction into account. Thus, the molar conductivity, diffusivity and cation transport number of the CuSO_4_ component decreases towards higher CuSO_4_ concentrations, whereas it is not affected by the Na_2_SO_4_ concentration. Likewise, the properties of the Na_2_SO_4_ component are influenced by its concentration and do not interact with the CuSO_4_ component. The modeled data is overestimating the measured limited current. At Na_2_SO_4_ concentrations below 0.05 M the differences between the modeled and measured data is with less than 5% moderate, however, it increases towards higher concentrations. As expected from the Debye–Hückel theory, the interaction of the ions is expected to decrease the molar conductivity and diffusion coefficients, explaining the modeled overestimation of the limited currents.
(iii) In the third parametrization scenario, the parameters of the CuSO_4_ and Na_2_SO_4_ components are calculated as a function of the total concentration of sulfate ions, assuming that the interaction between the sulfate ions and the different cations is equal. However, this parametrization underestimates the measured limited current. Thus, the assumption that the ions influence one another in the same amount does not hold valid. Figure 1 showed a more pronounced concentration dependence of molar conductivity, diffusion coefficient and transfer number of CuSO_4_ than those of Na_2_SO_4_. Thus, the ion–ion interactions in CuSO_4_ are different from that in Na_2_SO_4_ and the influence of the different components on one another is therefore also not that easy to describe.
(iv) The fourth case describes a concentration-dependent parameterization that displays a mixture between the second and third approach. Hereto, the parameters for the CuSO_4_ component are calculated for the concentration of CuSO_4_ plus 20% of the concentration of Na_2_SO_4_. Analogously, the parameters for the Na_2_SO_4_ component are calculated for the concentration of Na_2_SO_4_ plus 20% of the concentration of CuSO_4_. Thus, an interaction between the different ion types is partly included, however, to a smaller extent than that between the ions of the same type. Using this parameterization, a good fit of modeled and measured data is obtained.

The scenarios discussed above are based on the experimental data on binary electrolytes and do not represent a physicochemical approach to characterize the ion–ion interactions in mixed ion systems. Based on these data, however, the ion–ion interaction is shown to crucially impact the ion transport in supporting electrolytes and the related limited currents. In the literature^53^, the Debye–Hückel theory based physicochemical models that are used to describe the complex interaction between the ions are non-trivial and typically do not exactly describe the concentration dependence of the electrolyte parameters. However, using such approaches a physicochemical description of the ion–ion interactions for the electrolyte parameterization may be possible. Theoretical works to address the prediction of the ion–ion interaction in multi-ion systems have to follow and may lead to more precise parameterization procedures as the presented approaches that is based on the experimental data of binary electrolytic solutions.

### Computational sensibility analysis

Thus far, measured and modeled data showed that limited currents decrease towards higher concentrations of the inert ions. In the following, the modeled limited currents are modeled under a variation of the electrolyte parameters, aiming to display the physicochemical relations that are described by the differential equations of the transport model. Hereto, two cases are considered, a polar solvent in which the salts are dissolved and an ionic liquid (or ionic melt). The first case resembles the above discussed CuSO_4_–Na_2_SO_4_ system, where water displayed the polar solvent.

A simplified test system is considered in the following, in order to focus on the physicochemical interactions of the transport mechanisms rather than the complex parameterization that is discussed above. The test system is characterized by the following properties: (1) The electrolyte is comprised by two different salts, with two different types of cations (denoted as C1 and C2) and one type of anion (denoted as A). (2) Cation type C1 is pushed at the anode into the electrolyte and it is removed at the cathode, the other ions are inert. (3) Both salts have an equal density. (4) The molar conductivity, diffusion coefficient and transfer number of both salts are assumed as concentration independent with constant values of $\Lambda = 100 \,\text{ S }{\text{cm}}^{2} {\text{mol}}^{-1}$, $D={10}^{-5}\, {\text{cm}}^{2} {\text{s}}^{-1}$, ${t}^{+}=0.5$ and initial concentrations of $c=0.1\,\text{ M}$. One of these parameters of the supporting salt (that is constituted of C2 and A) are varied, while the other remain constant.

Figure 3 showed, that the net concentration of all ions at the cathode in the system decreases near the cathode. However, in the case of ionic liquids or salt melts, the depletion of the ions at an electrode means that the entire electrolyte vanishes whereas it concentrates at another electrode. For the test system with the ionic liquid an encapsulated volume is considered (such as in a battery). As both salts in the test system have the same type of anions and the same density, only the positions of the cations can change whereas the concentration of the anions is constant over the entire electrolyte. Hence, the differential equations that describe the anion transport are neglected and deleted from the source code.

**Figure 5:**
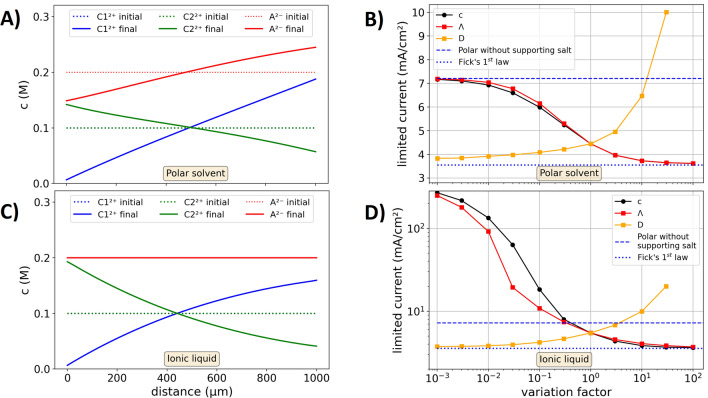
Modeled data to examine the influence of the electrolyte parameters on the limited current for an electrode distance of $0.1\, \text{cm}$, an initial concentration of $c=0.1\, \text{M}$, a molar conductivity $\Lambda =100\, \text{S}\, {\text{cm}}^{2}\, {\text{mol}}^{-1}$ and a diffusion coefficient of $D={10}^{-6}\, {\text{cm}}^{2}\,{\text{s}}^{-1}$ of the supporting salt and the reactive ion pair. (**A**) Concentration gradients obtained for the polar solvent. (**B**) Parameter variation for the polar solvent. The variation factor on the x-axis is multiplied to either the concentration, molar conductivity or diffusion coefficient of the supporting salt. Blue lines: Diffusion-limited current calculated by Fick’s first law and the limited current without addition of supporting salt. (**C,D**) Same as (**A**) and (**B**) but for an ionic liquid, where the ion displacement is confined as described in the text in more detail.

Figure 5A shows the modeled concentration gradients of the test system with a polar solvent. Despite the different parameterization, a similar shape of the concentration gradients compared to Fig. 3 is obtained. Figure 5B graphs the limited currents that are obtained under the parameter variation of the electrolyte with the polar solvent. With a negligible concentration or conductivity of the inert cations C2, the same limited current as in the case of the unsupported electrolyte is reached. With an increasing concentration or conductivity the limited current drops to the value described by Fick’s first law, in which diffusion is the only driving force. Thus, in this case the conduction of C1 has a minor impact on its net transport. Without the supporting salt, the limited current is approximately 2 times higher than that described by Fick’s first law. In the case of a variation factor of unity (equal properties of inert and active salt), the limited current is 1.25 higher than the diffusion-limited current.

In binary diluted electrolytes the conductivity does not influence the limited current, as the entire current is anyway carried by the ions^24^. However, as here defined by the conductivity ratio (Eq. 2), now the conductivity is decisive as it determines how much of the active ions C1 are actually carried by the conduction. The conductivity is shared between the different types of ions. When the conductivity of the inert ion C2 type becomes much larger than that of the active ion C1, the ion C1 is mainly transported by the driving force of the concentration gradient as the conduction is mainly done by C2. The limited current increases towards higher values of the diffusion coefficient of the inert salt. In this case, a depletion of ions at the cathode is avoided, from which also the limited current benefits.

Figure 5C shows the concentration gradient modeled for an ionic liquid. In this case, the amount of anions is constant over the distance (see discussion above). Figure 5D shows the modeled limited currents for the ionic liquid scenario. Without the supporting salt, the limited current is infinite as the boundary condition of the constant anion concentration does not allow a concentration gradient. With small additions of the supporting electrolyte, the supporting salt accumulates at the cathode and high concentration gradients of C1 over small distance close to the cathode result, for which the limited currents are orders of magnitude higher than those in the case of the polar solvent. Towards infinite concentrations or conductivities of C2, the limited current drops to the diffusion-limited current described by Fick’s first law (see reference^24^).

### Application of the results in electrochemical devices

The results on the copper based model system can find direct application in the design of electrochemical processes and devices such as copper refining^4,5^ or aqueous copper/sulfur batteries^55,56^. However, the aim of this article is to conclude physicochemical relations that are applicable to a wider scope and which are independent of the parameterization of the presented model system. With the knowledge that the limited current decreases with the amount of supporting ions, the addition of inert ions displays a compromise between an increased conductivity and a decreased limited current. When the electrolyte is mechanically mixed by convectional forces, the concentration gradients formed by the electrochemical current are partly equilibrated. Thus, the diffusion-limited currents in such flowing electrolytes is larger than in the case of static electrolytes. Causes for such convection can be found in: (i) Macroscopic density difference, where the gravitational force leads to shear forces. (ii) Bubble formation and ascending bubbles. (iii) Mechanical mixing of the electrolyte by stirring or pumping.

In ionic liquids, the electrode is always in contact with the electrolyte, which reduces with reference to Fig. 5 the depletion of the active ion type at the electrode in comparison to solvent based electrolytes. However, high viscosities typically cause smaller diffusion coefficients and conductivities of the active ion types in ionic liquids than that those of solvent based electrolytes. As a result, limited currents can display a severe limitation for electrochemical devices and processes that operate with ionic liquids. Further studies have to follow to experimentally examine limited currents in ionic liquids.

## Conclusion

This study examined the ion conduction, current-driven concentration gradients and related limited currents in supporting electrolytes. A computational model is developed to describe the ion transport and the related spatiotemporal ion concentrations in electrolytes with three ion types. This model is equipped with different concentration-dependent parameterization scenarios and evaluated with measured limited currents of CuSO_4_–Na_2_SO_4_ electrolytes. The comparison of measured and modeled data shows that a complex concentration-dependent parameterization of the interaction between the different ion types in supporting electrolytes is required to adequately model the ion transport. A computational study on the variation of the electrolyte parameters reveals the ion transport mechanisms and the interplay of electric-field and diffusion-driven ion motion. With an infinite amount of supporting salt in the electrolyte, the conduction-driven transport of the active ion becomes negligible and the limited current drops to the diffusion-limited current that is described by Fick’s first law. The similarities and differences of the ion transport in supporting electrolytes and ionic liquids is examined with the computational model, showing the impact of electrolyte parameters on limited currents.

## Supplementary Information

Supplementary Information.

## Data Availability

All data of the computational model generated or analysed during this study are included in this published article and its supporting information as it can be calculated and reproduced with the provided source code. The experimental datasets used and/or analysed during the current study are available from the corresponding author on reasonable request.
