# Supplementary material for: Ion transport and limited currents in supporting electrolytes and ionic liquids
Source: Sci Rep. 2022 Apr 13;12:6215. doi: 10.1038/s41598-022-10183-2 (PMC9008042; doi:10.1038/s41598-022-10183-2)
Supplement: Supplementary file 1 — Supplementary Information. [file 41598_2022_10183_MOESM1_ESM.docx]

**Supporting information on:**

**“Ion Transport and Limited Currents in Supporting**

**Electrolytes and Ionic Liquids”**

Maximilian Schalenbach*, Y. Emre Durmus, Hermann Tempel, Hans Kungl, Rüdiger-A. Eichel

Fundamental Electrochemistry (IEK‑9), Institute of Energy and Climate Research, Forschungszentrum Jülich GmbH, 52425 Jülich, Germany

# Equations of the ion transport model

The symmetry restrictions of the parallel electrodes alignment with equal surface areas allow to confine the model to one space dimension. The position in space is denoted by $x$ with $x = 0$ for the cathode and $x = d$ for the anode. The molar flux of the ions in space is denoted by$\Phi$, the current density as $j$, the conductivity by $\kappa$, the diffusion coefficient by $D$, the concentration by $c$ and the valence of ions by $z$. Superscript describes ion type (denoted by $i$), which is in this study to identify with $Cu^{2+}$ or $SO_{4}^{2-}$. Subscript equals either name of the process or the $x$-position in the discrete differential equation (denoted by $n$). The total amount of discrete space element is denoted by $n_{\mathrm{total}}$. The discrete difference between the space elements is described by $\Delta x=x/n_{\mathrm{total}}$.

Starting from the continuity equation, the spatiotemporal concentration of the $i$-th ion type is described by:

| $\frac{dc^{i}(x,t)}{dt}=-\frac{d\Phi^{i}\left( x,t \right)}{dx}$ | (1) |
| --- | --- |

The molar flux equals the sum of the contributions of diffusion $\Phi_{\mathrm{diff}}^{i}$ and electric field driven $\Phi_{\mathrm{el}}^{i}$ movement of the ions:

| $\frac{d\Phi^{i}\left( x,t \right)}{dx}=\frac{d\Phi_{\mathrm{diff}}^{i}\left( x,t \right)}{dx}+\frac{d\Phi_{\mathrm{el}}^{i}\left( x,t \right)}{dx}$ | (2) |
| --- | --- |

The term for the diffusion can be described by Fick’s second law:

| $\frac{d\Phi_{\mathrm{diff}}^{i}(x,t)}{dx}=D\frac{d^{2}c^{i}(x,t)}{dx^{2}}$ | (3) |
| --- | --- |

This equation can be discretized for the numerical model by

| $D\frac{d^{2}c^{i}(x,t)}{dx^{2}}\approx D^{i}\frac{c_{n+1}^{i}+c_{n-1}^{i}-{2c}_{n}^{i}}{\Delta x^{2}} ,$ | (4) |
| --- | --- |

where the numerical representation is an approximation of the analytical differential equation. On the basis of Faraday law, the molar flux attributable to the Ohmic conduction equals the current density divided by the Faraday constant $F$:

| $\frac{d\Phi_{\mathrm{el}}^{i}\left( x \right)}{dx}=\frac{1}{F z^{i}}\frac{dj^{i}}{dx}$ | (5) |
| --- | --- |

This equation can be discretized using a chain localized current densities along the discretized element $n$ of the considered electrolyte. The cations are coming from the anode to the cathode, so that the change in the *n-*th element of the cations is related to the *(n+1)-*th element:

| $\frac{dj_{n}^{i}}{dx}\approx\frac{j_{n+1}^{i}-j_{n}^{i}}{\Delta x}$ | (6) |
| --- | --- |

The anions are moving in the opposite direction:

| $\frac{dj_{n}^{i}}{dx}\approx\frac{j_{n}^{i}-j_{n+1}^{i}}{\Delta x}$ | (7) |
| --- | --- |

The localized current densities of the individual ion types in the bulk are described by

| $j_{n}^{i}=j \tilde{\kappa}_{n}^{i} ,$ | (8) |
| --- | --- |

where j denotes the overall current and $\tilde{\kappa}_{n}^{i}$ the conductivity ratio, which describes how much the ion $i$ contributes to the overall conductivity of the electrolyte in the spatial element *n.*

At the boundaries of the spatial domain, the electrochemical interfaces are described with $n=0$ for the cathode and$n=n_{\mathrm{total}}$ for the anode. Apparently, these spatial elements require a different description as that of the bulk electrolyte. Nevertheless, their input and output must be balanced to obey mass conservation which eases their description. At both interfaces the inert sulfate ions cannot be electrochemically converted, for which their currents are negligible: $j_{0}^{\mathrm{SO}_{4}^{2-}}=j_{0}^{\mathrm{Na}^{2+}}=0$ and $j_{n_{\mathrm{total}}}^{\mathrm{SO}_{4}^{2-}}=j_{n_{\mathrm{total}}}^{\mathrm{Na}^{2+}}=0$. Thus, the entire current must be carried by the copper ions $j_{0}^{Cu^{2+}}=j$ and $j_{n_{\mathrm{total}}}^{Cu^{2+}}=j$.

Equation 2 describes the differential equation for one ion type in the electrolyte. With a differential equation for every ion type a set of differential equations is build, which is numerically solved with the Euler forward algorithm. The electroneutrality must be locally maintained, as any change of it leads to an electric field that is instantaneously compensated by an electric field driven ion movement. The mathematical formulation of the local electroneutrality in one special element $n$ is given by:

| $\sum_{i} z_{n}^{i} c_{n}^{i}(x,t)=0$ | (9) |
| --- | --- |

After every step in time of the numerical simulation, any deviation from this equation is balanced by the conduction, while the movement of the different ion type is described by the conductivity ratio. Accordingly, the electroneutrality is implemented in the form of an instantaneous adjustment of the modeled concentrations. The solution are concentration matrices for each individual ion type denoted as $c^{i}(x,t)$. Such a matrix has a space ($x$) and time ($t$) dimension and includes one concentration value of the ion type $i$ for every increment in the spatiotemporal framework considered.

# Fit of the electrolyte properties

The literature data on the electrolyte properties presented in Figure 1 of the article showed fits that describe the concentration dependence of the molar conductivities and diffusion coefficients by means of a mathematical analytical and continuous expression. The molar conductivities $\Lambda$ as a function of the concentration $c$ were fitted with the equation

| $\Lambda=\alpha-\beta c^{0.005}+\gamma c^{0.001}$ | (10) |
| --- | --- |

while the diffusion coefficients were fitted with the equation

| $D=\left( -\epsilon c^{\zeta}+\eta\right)\times{10}^{-5}$ | (11) |
| --- | --- |

where $\alpha$, $\beta$, $\gamma$,$\epsilon$,$\zeta$ and $\eta$ represent fit parameters. The values of these fit parameters are summarized in Table S1.

Table S1: Fit parameters for equation 10 and 11

|  | $\alpha$ | $\beta$ | $\gamma$ | $\epsilon$ | $\zeta$ | $\eta$ |
| --- | --- | --- | --- | --- | --- | --- |
| CuSO_4_ | 543243.2 | -132051.6 | -675248.3 | 4.7464 | 0.2336 | 8.5 |
| Na_2_SO_4_ | -219216.4 | 63298.4 | 282606.9 | 0.4896 | 0.4598 | 1.15 |

# Implementation of the Ion Transport Model

In the following, the source codes of the ion transport model are presented. The codes is written in Python 3 and runs on a state of the art personal computer without any advanced hardware requirements.

## Codes to parameterize the electrolyte

import numpy as np

import pandas as pd

import matplotlib.pyplot as plt

import os

from scipy.optimize import curve_fit

# =============================================================================

# Style figures

# =============================================================================

import matplotlib

matplotlib.rc('xtick', labelsize=16)

matplotlib.rc('ytick', labelsize=16)

font1 = {'family' : 'normal',

'weight' : 'normal',

'size' : 18}

matplotlib.rc('font', **font1)

matplotlib.rcParams["figure.figsize"] = (7.5,5)

# =============================================================================

class electrolyte_properties:

"""

When concentration passed via "submit_concentration", then the electrolyte properties

are recalculated and stored in dictionaries

"""

def __init__(self):

# all correction factors initially defined as 1

# Can be changed within the main code

self.savepath_pics = "electrolyte_pics"

self.cf_CuSO4_mc = 1

self.cf_CuSO4_D = 1

self.cf_CuSO4_t = 1

self.cf_Na2SO4_mc = 1

self.cf_Na2SO4_D = 1

self.cf_Na2SO4_t = 1

self.exp = 0.001

# graph properties

self.x_min = 6e-3

self.x_max = 2

self.mc_max = 250

self.mc_min = 25

self.mc_txt = 30

self.D_min = 2.5

self.D_max = 12

self.D_txt = 2.8

self.t_max = 0.41

self.t_min = 0.24

self.t_txt = 0.245

self.txt_box_props = dict(boxstyle='round', facecolor='wheat', alpha=0.5)

# Dictionaries with stored information

self.c = {

"CuSO4" : 0,

"Na2SO4" : 0

}

self.mc = {}

self.D = {}

self.t = {}

# Dictionary with the stored information of dummy ions

self.mc0 = {}

self.D0 = {}

self.t0 = {}

# initialize the fits

self.CuSO4_fit()

self.Na2SO4_fit()

# initialize the dummy classes

self.active_ions_init()

self.supporting_ions_init()

# =============================================================================

# Concentration Stuff

# =============================================================================

def submit_concentration(self,species, c):

# c is a float or a numpy array

self.c[species] = c*1000 # factor 100 to convert from mol/cm³ to mol/l

if species =="CuSO4":

self.CuSO4_mc()

self.CuSO4_D()

self.CuSO4_t()

elif species =="Na2SO4":

self.Na2SO4_mc()

self.Na2SO4_D()

self.Na2SO4_t()

elif species == "active_ions":

self.active_ions_mc()

self.active_ions_D()

self.active_ions_t()

elif species == "supporting_ions":

self.supporting_ions_mc()

self.supporting_ions_D()

self.supporting_ions_t()

else:

print("species not known")

# =============================================================================

# CuSO4

# =============================================================================

def CuSO4_mc(self):

self.mc["CuSO4"] = self.cf_CuSO4_mc*(self.params_CuSO4_mc[0] - self.params_CuSO4_mc[1]*self.c["CuSO4"]**0.005 + self.params_CuSO4_mc[2]*self.c["CuSO4"]**0.001)

def CuSO4_D(self):

self.D["CuSO4"] = self.cf_CuSO4_D*(-self.params_CuSO4_D[0]*self.c["CuSO4"]**self.params_CuSO4_D[1]+self.params_CuSO4_D[2])*1e-6

def CuSO4_t(self):

self.t["CuSO4"] = self.cf_CuSO4_t*(self.c["CuSO4"]*0+1)*(0.401-0.1426*self.c["CuSO4"]**(0.5) + 0.0186*self.c["CuSO4"])

def CuSO4_fit(self):

"""

Detemrines the parameters of the fits

"""

# =============================================================================

# CuSO4 : fit to concentration dependence of the conductivity

# =============================================================================

# Data from OWEN and GURRY

c1 = np.asarray([4.95E-04,2.05E-03,5.03E-03,8.85E-03,1.30E-02,1.78E-02,2.42E-02,3.17E-02,3.99E-02,4.72E-02,5.57E-02,6.66E-02,7.74E-02,9.74E-02,1.15E-01])

kappa1 = np.asarray([232,195,166,148,137,127,119,112,106,102,98.6,94.8,91.7,87.2,84])

# data from Bester-Roag @25°C

c2 = np.asarray([0.02,0.04,0.06,0.08,0.1,0.12,0.14,0.16,0.18,0.2,0.25,0.3,0.35,0.4,0.45,0.5,0.55,0.6,0.65,0.7,0.75,0.8,0.85,0.9,0.95,1,1.05,1.1,1.15,1.2,1.25,1.3,1.35,1.4,1.45,1.5])

kappa2 = np.asarray([124.2,107.0,98.0,92.0,87.6,84.2,81.3,78.9,76.8,75.0,71.1,68.1,65.5,63.3,61.3,59.5,57.9,56.4,55.0,53.7,52.4,51.2,50.1,49.0,47.9,46.9,45.9,44.9,44.0,43.0,42.1,41.2,40.3,39.5,38.6,37.8])

# data from Bester-Roag @ 20°C

kappa3 = np.asarray([112.6,97.1,88.9,83.6,79.6,76.4,73.8,71.6,69.7,68.1,64.6,61.8,59.4,57.4,55.6,54.0,52.5,51.1,49.8,48.6,47.4,46.3,45.3,44.2,43.3,42.3,41.4,40.5,39.6,38.7,37.9,37.0,36.2,35.4,34.6,33.8])

# merge data

c_25 = np.append(c1,c2)

kappa_25 = np.append(kappa1,kappa2)

# fit functions

def cond_fct(x,a,b,c):

return a - b*x**0.005 + c*x**0.001

def cond_fct_fit(x,y):

print(x)

popt, pcov = curve_fit(cond_fct, x, y)#, bounds=([0,0.1],[100,1.2]))

return popt

self.params_CuSO4_mc = cond_fct_fit(c_25,kappa_25)

# plot fit data

x = np.arange(0.001,1.55,0.002)

self.c["CuSO4"] = x

plt.scatter(c1,kappa1, color ="red", label = "Owen et al. @ 25°C")

plt.scatter(c2,kappa2, color ="grey", label = "Bester-Roag et al. @ 25°C")

plt.scatter(c2,kappa3, color ="grey", marker = "x",label = "Bester-Roag et al. @ 20°C")

#plt.scatter(c3,kappa3, label = "CRC")

self.CuSO4_mc()

plt.plot(self.c["CuSO4"],self.mc["CuSO4"], "k", label = "fit @ 25°C")

plt.plot(self.c["CuSO4"],0.91*self.mc["CuSO4"], "k:", label = "fit @ 20°C")

#plt.plot(x,kappa_c(x), "k", label = "fit Owen et.al.")

plt.xlabel("c (M)")

plt.ylabel("$\Lambda }$ (S cm² mol $^{-1}$)")

plt.legend(prop={'size': 14})

plt.grid()

plt.xscale("log")

plt.xlim(self.x_min,self.x_max)

plt.text(self.x_min+0.01, self.mc_txt, "CuSO$_{4}$", fontsize=18, verticalalignment='bottom', bbox=self.txt_box_props)

plt.ylim(self.mc_min,self.mc_max)

plt.savefig(self.savepath_pics+"/CuSO4_mc.png", bbox_inches = "tight", dpi = 200)

plt.show()

# =============================================================================

# CuSO4 : fit to concentration dependence of the diffusion

# =============================================================================

# Data of Emanuel

c_1 = np.asarray([0,0.1,0.2,0.35,0.4])

D_1 = np.asarray([8.5,5.6,5.4,5.1,4.86])

# Data of Noulty

c_2 = np.asarray([0.00269,0.00544,0.0108,0.0266,0.0468,0.054,0.0598,0.0738,0.0818,0.0997,0.117,0.16,0.199,0.214,0.266,0.337,0.388,0.481,0.52,0.618,0.708,0.814,0.924])

D_2 = np.asarray([0.718,0.694,0.672,0.629,0.616,0.602,0.609,0.592,0.583,0.577,0.559,0.547,0.534,0.514,0.498,0.475,0.471,0.452,0.433,0.421,0.39,0.389,0.379])*10

# Data of Woolf

c_3 = np.asarray([0,0.01,0.04,0.09,0.16,0.25])

D_3 = np.asarray([0.858,0.713,0.632,0.587,0.547,0.513])*10

# merged data

c_all = np.append(np.append(c_1,c_2),c_3)

D_all = np.append(np.append(D_1,D_2),D_3)

#offset = 1

def power_fct(x,a,b):

return -a*x**b+offset

def power_fct_fit(x,y):

popt, pcov = curve_fit(power_fct, x, y, bounds=([0,0.1],[100,1.2]))

#fit = func(x, *popt)

return popt

offset = 8.5

self.params_CuSO4_D = np.append(power_fct_fit(c_all,D_all),offset)

self.c["CuSO4"] = np.arange(0,1.55,0.002)

plt.scatter(c_1,D_1, label = "Emanuel et.al.")

plt.scatter(c_2,D_2, label = "Noulty et.al.")

plt.scatter(c_3,D_3, label = "Woolf et.al.")

self.CuSO4_D()

plt.plot(self.c["CuSO4"],self.D["CuSO4"]*1e6, "k", label = "fit @ 25°C")

plt.plot(self.c["CuSO4"],0.88*self.D["CuSO4"]*1e6, "k:", label = "fit @ 20°C")

plt.legend(prop={'size': 14})

plt.xlabel("c (M)")

plt.ylabel("D (10"+"$^{-6}$ cm² s$^{-1}$)")

plt.grid()

plt.xscale("log")

plt.xlim(self.x_min,self.x_max)

plt.ylim(self.D_min,self.D_max)

plt.text(self.x_min+0.01, self.D_txt, "CuSO$_{4}$", fontsize=18, verticalalignment='bottom', bbox=self.txt_box_props)

plt.savefig(self.savepath_pics+"/CuSO4_D.png", bbox_inches = "tight", dpi = 200)

plt.show()

# =============================================================================

# CuSO4 : fit to concentration dependence of the transfer number

# =============================================================================

# Data of Pikal

self.CuSO4_t()

plt.plot(self.c["CuSO4"],self.t["CuSO4"], color = "b", label = "Pikal et al.")

plt.legend(prop={'size': 14})

plt.xlabel("c (M)")

plt.ylabel("t$^{+}$")

plt.grid()

plt.xscale("log")

plt.xlim(self.x_min,self.x_max)

plt.ylim(self.t_min,self.t_max)

plt.text(self.x_min+0.01, self.t_txt, "CuSO$_{4}$", fontsize=18, verticalalignment='bottom', bbox=self.txt_box_props)

plt.savefig(self.savepath_pics+"/CuSO4_f.png", bbox_inches = "tight", dpi = 200)

plt.show()

print("Fitted and loaded CuSO4 data")

# =============================================================================

# NaSO4

# =============================================================================

def Na2SO4_mc(self):

self.mc["Na2SO4"] = (self.cf_Na2SO4_mc*(self.params_Na2SO4_mc[0] - self.params_Na2SO4_mc[1]*self.c["Na2SO4"]**0.005 + self.params_Na2SO4_mc[2]*self.c["Na2SO4"]**0.001)).clip(0,240)

def Na2SO4_D(self):

self.D["Na2SO4"] = (-self.params_Na2SO4_D[0]*self.c["Na2SO4"]**self.params_Na2SO4_D[1]+self.params_Na2SO4_D[2])*1e-5

def Na2SO4_t(self):

self.t["Na2SO4"] = (self.params_Na2SO4_t[0]*self.c["Na2SO4"]**self.params_Na2SO4_t[1]+self.params_Na2SO4_t[2]).clip(0,0.385)

def Na2SO4_fit(self):

# =============================================================================

# CuSO4 : fit to concentration dependence of the conductivity

# =============================================================================

# Data from OWEN and GURRY

c = np.asarray([0.01, 0.02, 0.05, 0.1, 0.2, 0.5, 1])

molar_cond = np.asarray([225.16, 231.98, 182.98, 163.08, 142.59, 117.73, 92.33])

# fit functions

def cond_fct(x,a,b,c):

return a - b*x**0.005 + c*x**0.001

def cond_fct_fit(x,y):

print(x)

popt, pcov = curve_fit(cond_fct, x, y)#, bounds=([0,0.1],[100,1.2]))

return popt

self.params_Na2SO4_mc = cond_fct_fit(c,molar_cond)

# plot fit data

x = np.arange(0.001,1.55,0.002)

self.c["Na2SO4"] = x

plt.scatter(c,molar_cond,label = "Measured @ 20°C")

#plt.scatter(c3,kappa3, label = "CRC")

self.Na2SO4_mc()

plt.plot(self.c["Na2SO4"],self.mc["Na2SO4"], "k:", label = "fit @ 20°C")

#plt.plot(x,kappa_c(x), "k", label = "fit Owen et.al.")

plt.xlabel("c (M)")

plt.ylabel("$\Lambda }$ (S cm² mol $^{-1}$)")

plt.legend(prop={'size': 14})

plt.grid()

plt.xscale("log")

plt.xlim(self.x_min,self.x_max)

plt.ylim(self.mc_min,self.mc_max)

plt.text(self.x_min+0.01, self.mc_txt, "Na$_{2}$SO$_{4}$", fontsize=18, verticalalignment='bottom', bbox=self.txt_box_props)

plt.savefig(self.savepath_pics+"/Na2SO4_mc.png", bbox_inches = "tight", dpi = 200)

plt.show()

# =============================================================================

# NaSO4: fit to concentration dependence of the diffusion

# =============================================================================

# Data of Rard

c= [0.004984,0.007465,0.009966,0.019931,0.039288,0.074681,0.099528,0.14913,0.19872,0.24813,0.35653,0.48399,0.62984,0.79322,0.97414,1.1712,1.3405,1.4033,1.5282]

D= [1.1204,1.1033,1.09,1.06,1.0352,1.0002,0.9798,0.9501,0.9224,0.8978,0.8488,0.7978,0.748,0.702,0.6578,0.6198,0.5906,0.5824,0.5645]

def power_fct(x,a,b):

return -a*x**b+offset

def power_fct_fit(x,y):

popt, pcov = curve_fit(power_fct, x, y)#, bounds=([0,0.1],[100,1.2]))

#fit = func(x, *popt)

return popt

offset = 1.15

self.params_Na2SO4_D = np.append(power_fct_fit(c,D),offset)

# fit functions

# plot fits

self.c["Na2SO4"] = np.arange(0,1.55,0.002)

plt.scatter(c,np.asarray(D)*10, label = "Rard et.al.")

self.Na2SO4_D()

plt.plot(self.c["Na2SO4"],self.D["Na2SO4"]*1e6, "k", label = "fit @ 25°C")

plt.plot(self.c["Na2SO4"],0.88*self.D["Na2SO4"]*1e6, "k:", label = "fit @ 20°C")

plt.legend(prop={'size': 14})

plt.xlabel("c (M)")

plt.ylabel("D (10"+"$^{-5}$ cm² s$^{-1}$)")

plt.grid()

plt.xscale("log")

plt.xlim(self.x_min,self.x_max)

plt.ylim(self.D_min,self.D_max)

plt.text(self.x_min+0.01, self.D_txt, "Na$_{2}$SO$_{4}$", fontsize=18, verticalalignment='bottom', bbox=self.txt_box_props)

plt.savefig(self.savepath_pics+"/Na2SO4_D.png", bbox_inches = "tight", dpi = 200)

plt.show()

# =============================================================================

# NaSO4: fit to concentration dependence of the transfer number

# =============================================================================

# Data of

c = [0.009931, 0.019808, 0.050347, 0.100125, 0.19824]

t = [0.3848, 0.3836, 0.3829, 0.3828, 0.3828]

self.c["Na2SO4"] = np.arange(0.000001,1.55,0.002)

offset = 0.38275

def power_fct_t(x,a,b):

return a*x**b+offset

def power_fct_fit_t(x,y):

popt, pcov = curve_fit(power_fct_t, x, y, bounds=([0,-2],[100,0]))

#fit = func(x, *popt)

return popt

self.params_Na2SO4_t = np.append(power_fct_fit_t(c,t),offset)

plt.scatter(c,t, label = "Longsworth")

self.Na2SO4_t()

plt.plot(self.c["Na2SO4"],self.t["Na2SO4"], "k", label = "fit")

plt.legend(prop={'size': 14},loc = "right")

plt.xlabel("c (M)")

plt.ylabel("t$^{+}$")

plt.grid()

plt.xscale("log")

plt.xlim(self.x_min,self.x_max)

plt.ylim(self.t_min,self.t_max)

plt.text(self.x_min+0.01, self.t_txt, "Na$_{2}$SO$_{4}$", fontsize=18, verticalalignment='bottom', bbox=self.txt_box_props)

plt.savefig(self.savepath_pics+"/Na2SO4_t.png", bbox_inches = "tight", dpi = 200)

plt.show()

print("Fitted and loaded Na2SO4 data")

# =============================================================================

# # For modeling of the concentration variation

# =============================================================================

def active_ions_init(self):

# Default values of the parameters

self.mc0["active_ions"] = 100

self.D0["active_ions"] = 1e-5

self.t0["active_ions"] = 0.5

def active_ions_mc(self):

self.mc["active_ions"] = self.c["active_ions"]*0 + self.mc0["active_ions"]

def active_ions_D(self):

self.D["active_ions"] = self.c["active_ions"]*0 + self.D0["active_ions"]

def active_ions_t(self):

self.t["active_ions"] = self.c["active_ions"]*0 + self.t0["active_ions"]

def supporting_ions_init(self):

# Default values of the parameters

self.mc0["supporting_ions"] = 100

self.D0["supporting_ions"] = 1e-5

self.t0["supporting_ions"] = 0.5

def supporting_ions_mc(self):

self.mc["supporting_ions"] = self.c["supporting_ions"]*0 + self.mc0["supporting_ions"]

def supporting_ions_D(self):

self.D["supporting_ions"] = self.c["supporting_ions"]*0 + self.D0["supporting_ions"]

def supporting_ions_t(self):

self.t["supporting_ions"] = self.c["supporting_ions"]*0 + self.t0["supporting_ions"]

# =============================================================================

# e = electrolyte_properties()

# e.submit_concentration("CuSO4",1e-4)

# print(e.c["CuSO4"])

# print(e.CuSO4_mc())

# =============================================================================

## Main Code

import numpy as np

import pandas as pd

import matplotlib.pyplot as plt

import os

from scipy.optimize import curve_fit

# =============================================================================

# Style figures

# =============================================================================

import matplotlib

matplotlib.rc('xtick', labelsize=16)

matplotlib.rc('ytick', labelsize=16)

font1 = {'family' : 'normal',

'weight' : 'normal',

'size' : 18}

matplotlib.rc('font', **font1)

matplotlib.rcParams["figure.figsize"] = (7.5,5)

# =============================================================================

# import electrolyte properties class

import electrolyte_properties

E = electrolyte_properties.electrolyte_properties()

def closest_index(index_list, time):

"""

finds the index of the df, that is closest to the the time

Used to implement the experimetnally detemrined current profile

"""

np_index = np.asarray(index_list)

return np_index[np.argmin(abs(np_index-time))]

def model(savepath, distance, x_steps, salt_1, c_C1_0, salt_2, c_C2_0, time, path_j_profile):

"""

calculate spatiotemporal ion concentrations

"""

if current_profile == True:

###### CURRENT PROFILE ###########################################

df_current_profile = pd.read_csv(path_j_profile)

df_current_profile = df_current_profile.set_index("time")

###### CURRENT PROFILE ###########################################

# Time and Space resolution

time_steps = 10000 #Anzahl iterationen

F = 96485 #C/mol

# Plotting resolution

t_res = 1 # time resolution in seconds

t_unit = "s"

x_res = 10*1000 # spatial resolution in micrometer

x_unit = "µm"

# Cations

species_C1 = 'Cu\N{SUPERSCRIPT TWO}⁺'

c_C1_0_mol_l = c_C1_0 # mol/l

c_C1_0 = c_C1_0_mol_l/1000 # mol/cm³ = 1/1000 mol/l

z_C1 = 2

bound_ca_C1 = 1

bound_an_C1 = 1

species_C2 = '2Na⁺'

c_C2_0_mol_l = c_C2_0 # mol/l

c_C2_0 = c_C2_0_mol_l/1000 # mol/cm³ = 1/1000 mol/l

z_C2 = 2

bound_ca_C2 = 0

bound_an_C2 = 0

# Anions

species_S = 'SO\N{SUBSCRIPT FOUR}\N{SUPERSCRIPT TWO}⁻'

z_A1 = 2

c_A1_0 = (z_C1*c_C1_0+z_C2*c_C2_0)/(z_A1) # mol/cm³ = 1/1000 mol/l

bound_ca_A1 = 0

bound_an_A1 = 0

# Discritization of the model

x_stepsh = x_steps +1

time_stepsh = time_steps+1

dx = distance/x_steps

dt = time/time_steps

# =============================================================================

# Concentration: Initial and definitions

# =============================================================================

# Define concentration matrix: x-Axis: distance, y-axis: time

c_C1 = np.zeros((time_stepsh,x_stepsh))

c_C2 = np.zeros((time_stepsh,x_stepsh))

c_A1 = np.zeros((time_stepsh,x_stepsh))

# Defininition of vectors that save the incremental time step

c_C1_t = np.zeros((x_stepsh))

c_C2_t = np.zeros((x_stepsh))

c_A1_t = np.zeros((x_stepsh))

# Definition of current aray

current_array = np.zeros((time_stepsh))

kappa_marix = np.zeros((time_stepsh,x_stepsh))

# Vector saving the differences in comparison to electroneutrality

c_charge_diff= [0 for x in range(x_stepsh)]

# Define initial values

for i in range(0, x_stepsh, 1):

# constant concentration profile:

c_C1[0][i]= c_C1_0

c_C2[0][i]= c_C2_0

c_A1[0][i]= c_A1_0

# Mean values of the concentration profiles

# Relevant is concentration gradient

c_C1_0m = np.mean(c_C1[0][:])

c_C2_0m = np.mean(c_C2[0][:])

c_A1_0m = np.mean(c_A1[0][:])

# console output

print(' ')

print('initial concentrations')

print(np.mean(c_C1_0m))

print(np.mean(c_C2_0m))

print(np.mean(c_A1_0m))

cond_ratio_C1 = np.zeros((x_stepsh))

cond_ratio_C2 = np.zeros((x_stepsh))

cond_ratio_A1 = np.zeros((x_stepsh))

# =============================================================================

# Further Definitions

# =============================================================================

# conductivity ratios

def f_cond_ratio_C1(c1,c2):

return E.t[salt_1]*E.mc[salt_1]*c1/(E.mc[salt_1]*c1 + E.mc[salt_2]*c2)

def f_cond_ratio_C2(c1,c2):

return E.t[salt_2]*E.mc[salt_2]*c2/(E.mc[salt_1]*c1 + E.mc[salt_2]*c2)

def f_cond_ratio_A1(c1,c2):

return ( (1-E.t[salt_1])*E.mc[salt_1]*c1 + (1-E.t[salt_2])*E.mc[salt_2]*c2 )/(E.mc[salt_1]*c1 + E.mc[salt_2]*c2)

# =============================================================================

# time iteration of concentration profiles

# =============================================================================

if current_profile == False:

current = start_current

for t in range(0, time_steps, 1):

if current_profile == True:

###### CURRENT PROFILE ###########################################

index = closest_index(list(df_current_profile.index), t)

current = df_current_profile.at[index,"j"]/1000#*0.95

###### CURRENT PROFILE ###########################################

# submit concentrations to the Electrolyte_properties class

# Hier ist die Annahme, dass die geminse Konzentration die Konzentrationsabhängigkeit vorgibt!!!

if interaction == "none":

# no interaction at all

E.submit_concentration(salt_1, c_C1[0][:])

E.submit_concentration(salt_2, c_C2[0][:])

elif interaction == "individual":

# just interaction between same ion types

E.submit_concentration(salt_1, c_C1[t][:])

E.submit_concentration(salt_2, c_C1[t][:])

elif interaction == "combined":

# concentrations similarly influence the eletrolyte properties

E.submit_concentration(salt_1, c_C1[t][:]+c_C2[t][:])

E.submit_concentration(salt_2, c_C1[t][:]+c_C2[t][:])

elif interaction == "combined_20":

# concentrations similarly influence the eletrolyte properties

E.submit_concentration(salt_1, c_C1[t][:]+0.2*c_C2[t][:])

E.submit_concentration(salt_2, 0.2*c_C1[t][:]+c_C2[t][:])

else:

print("no interaction type defined")

break

# calculate conductivty ratios

cond_ratio_C1 = f_cond_ratio_C1(c_C1[t][:],c_C2[t][:])

cond_ratio_C2 = f_cond_ratio_C2(c_C1[t][:],c_C2[t][:])

cond_ratio_A1 = f_cond_ratio_A1(c_C1[t][:],c_C2[t][:])

# calcluate diffusion vectors

D_vec_C1 = E.D[salt_1]

D_vec_C2 = E.D[salt_2]

# Determine the diffusion coefficient of the anion by the concentration weighted middle of both salts

D_vec_A1C1 = E.D[salt_1]

D_vec_A1C2 = E.D[salt_2]

D_vec_A1 = (D_vec_A1C1*c_C1[t][:]+D_vec_A1C2*c_C2[t][:])/(c_C1[t][:]+c_C2[t][:])

# =============================================================================

# Wie soll man vernünftige werte !!!

# =============================================================================

# Calculate boundary values for the cathode at x = 0

c_C1_t[0] = (current/(z_C1*F*dx)*(-bound_ca_C1 + cond_ratio_C1[1]) + D_vec_C1[0]/(dx**2)*(-c_C1[t][0]+c_C1[t][1]))*dt + c_C1[t][0]

c_C2_t[0] = (current/(z_C2*F*dx)*(-bound_ca_C2 + cond_ratio_C2[1]) + D_vec_C2[0]/(dx**2)*(-c_C2[t][0]+c_C2[t][1]))*dt + c_C2[t][0]

c_A1_t[0] = (current/(z_A1*F*dx)*(+bound_ca_A1 - cond_ratio_A1[1]) + D_vec_A1[0]/(dx**2)*(-c_A1[t][0]+c_A1[t][1]))*dt + c_A1[t][0]

# Calculate boundary values for the anode x = xsteps

c_C1_t[x_steps] = (current/(z_C1*F*dx)*( bound_an_C1 - cond_ratio_C1[x_steps]) + D_vec_C1[x_steps]/(dx**2)*(-c_C1[t][x_steps]+c_C1[t][x_steps-1]))*dt + c_C1[t][x_steps]

c_C2_t[x_steps] = (current/(z_C2*F*dx)*( bound_an_C2 - cond_ratio_C2[x_steps]) + D_vec_C2[x_steps]/(dx**2)*(-c_C2[t][x_steps]+c_C2[t][x_steps-1]))*dt + c_C2[t][x_steps]

c_A1_t[x_steps] = (current/(z_A1*F*dx)*(-bound_an_A1 + cond_ratio_A1[x_steps]) + D_vec_A1[x_steps]/(dx**2)*(-c_A1[t][x_steps]+c_A1[t][x_steps-1]))*dt + c_A1[t][x_steps]

# iteration between boundaries

for i in range (1,x_steps,1):

c_C1_t[i] = (current/(z_C1*F*dx)*( cond_ratio_C1[i+1] - cond_ratio_C1[i]) + D_vec_C1[i]/(dx**2)*(-2*c_C1[t][i]+ c_C1[t][i+1]+ c_C1[t][i-1]))*dt + c_C1[t][i]

c_C2_t[i] = (current/(z_C2*F*dx)*( cond_ratio_C2[i+1] - cond_ratio_C2[i]) + D_vec_C2[i]/(dx**2)*(-2*c_C2[t][i]+ c_C2[t][i+1]+ c_C2[t][i-1]))*dt + c_C2[t][i]

c_A1_t[i] = (current/(z_A1*F*dx)*(-cond_ratio_A1[i+1] + cond_ratio_A1[i]) + D_vec_A1[i]/(dx**2)*(-2*c_A1[t][i]+ c_A1[t][i+1]+ c_A1[t][i-1]))*dt + c_A1[t][i]

# START 2nd order correction #####################################

# remain electroneutrality

#re-calculate updated conductivity ratios

cond_ratio_C1 = f_cond_ratio_C1(c_C1_t,c_C2_t)

cond_ratio_C2 = f_cond_ratio_C2(c_C1_t,c_C2_t)

cond_ratio_A1 = f_cond_ratio_A1(c_C1_t,c_C2_t)

# Compensate any diffference to electro neutrality with conduction

for i in range (0,x_stepsh,1):

c_charge_diff[i] = z_C1*c_C1_t[i]+z_C2*c_C2_t[i] - z_A1*c_A1_t[i]

c_C1_t[i] = (c_C1_t[i] - c_charge_diff[i]*cond_ratio_C1[i]/z_C1)

c_C2_t[i] = (c_C2_t[i] - c_charge_diff[i]*cond_ratio_C2[i]/z_C2)

c_A1_t[i] = (c_A1_t[i] + c_charge_diff[i]*cond_ratio_A1[i]/z_A1)

# Overall Amount of ions must be constant

c_C1_tm = np.mean(np.asarray(c_C1_t))

c_C2_tm = np.mean(np.asarray(c_C2_t))

c_A1_tm = np.mean(np.asarray(c_A1_t))

for i in range (0,x_stepsh,1):

c_C1_t[i] = c_C1_t[i] + (c_C1_0m - c_C1_tm)

c_C2_t[i] = c_C2_t[i] + (c_C2_0m - c_C2_tm)

c_A1_t[i] = c_A1_t[i] + (c_A1_0m - c_A1_tm)

# End 2nd order correction#########################################################

# Recursive overtaking values

for i in range (0,x_stepsh,1):

c_C1[t+1][i] = c_C1_t[i]

c_C2[t+1][i] = c_C2_t[i].clip(0,)

c_A1[t+1][i] = c_A1_t[i]

# Break if no more copper ions at the cathode

if (c_C1_t[0]<c_C1_0m/100):

print("exceeded minimum concentration")

break

if limiting_current == True:

if c_C1[t+1][0] < c_C1_0m/12:

if c_C1[t+1][0] < c_C1_0m/15:

current = current - 1e-5

else:

current = current + 1e-5

if c_C1[t+1][0] < c_C1_0m/20: # rapid change to avoid depletion

current = current - 5e-4

current_array[t] = current

#

print("")

print("diffusion limited current is: "+str(current))

print("")

df_current = pd.DataFrame()

print(len(current_array))

df_current["t"] = np.arange(0,t+2,1)*dt/t_res

df_current["j"] = current_array[:len(df_current)]

df_current = df_current.drop(df_current.tail(1).index)

df_current.to_csv(savepath+"/df_current.csv", index = False)

# =============================================================================

# Define DataFrame for further data evaluation

# =============================================================================

# make DataFrames of concentration matrices

c_C1 = c_C1[:t][:]

c_C2 = c_C2[:t][:]

c_A1 = c_A1[:t][:]

kappa_marix = kappa_marix[:t][:]

dfC1 = pd.DataFrame(c_C1)

dfC2 = pd.DataFrame(c_C2)

dfA1 = pd.DataFrame(c_A1)

dfkappa = pd.DataFrame(kappa_marix)

t_total = t*dt

print("total time = "+str(t_total))

#index = time in sec

time_array = np.arange(0,t,1)*dt/t_res

dfC1 = dfC1.set_index(time_array).rename_axis('time')

dfC2 = dfC2.set_index(time_array).rename_axis('time')

dfA1 = dfA1.set_index(time_array).rename_axis('time')

dfkappa = dfkappa.set_index(time_array).rename_axis('time')

# columns = distance in mm

x_array = np.round(np.arange(0,x_steps+1,1)*distance*x_res/x_steps,2)

dict_names = dict(zip(np.array(list(dfC1)).astype(int),x_array.astype(int).tolist()))

dfC1 = dfC1.rename(dict_names, axis='columns')

dfC2 = dfC2.rename(dict_names, axis='columns')

dfA1 = dfA1.rename(dict_names, axis='columns')

dfkappa = dfkappa.rename(dict_names, axis='columns')

dfC1.iloc[::100, :].to_csv(savepath+'//dfC1.csv',sep = ";")

dfC2.iloc[::100, :].to_csv(savepath+'//dfC2.csv',sep = ";")

dfA1.iloc[::100, :].to_csv(savepath+'//dfA1.csv',sep = ";")

# =============================================================================

# Calculate resistance

# =============================================================================

#calculate conductance matrix:

conductance_matrix = (1/dx)*dfkappa # Funktioniert das wirklich???????????????????????????????????????????????????????????????????????????

# unity matrice

unity = dfC1*0+1

#convert to resistance matrix and convert pandas dataframe to numpy array

resistance_matrix = (unity/conductance_matrix).values

#resistance over time

resistance_total = np.sum(resistance_matrix, axis = 1)

df_t = pd.DataFrame()

df_t["time"] = time_array

df_t["Resistance"] = resistance_total

df_t["Ohmic_drop"] = resistance_total*current

df_t = df_t.set_index("time")

df_t.iloc[::100, :].to_csv(savepath+'//dft.csv')

# =============================================================================

# Plotting

# =============================================================================

print("")

print("Start plotting")

print("")

time_series=[0,0.25,0.5,0.75,1]

print("Current over time")

plt.plot(df_current["t"], df_current["j"]*1000, 'k',linewidth=2)

plt.xlabel('time (s)')

plt.ylabel("current density (mA/cm²)")

plt.legend(prop={'size': 14})

plt.savefig(savepath+'//current.png', bbox_inches = "tight", dpi = 200)

plt.show()

print("Resistance over time")

plt.plot(df_t.index, df_t["Resistance"], 'k', label = "R$_{area}$ modeled",linewidth=2)

#plt.title('current = '+str(current*1000)+' mA/cm²')

plt.xlabel('time (s)')

plt.ylabel("area resistance (Ω cm²)")

plt.legend(prop={'size': 14})

plt.savefig(savepath+'//resistance.png', bbox_inches = "tight", dpi = 200)

plt.show()

print(' initial and final concentrations ')

plt.plot(x_array, 1000*dfC1.iloc[0], 'b:',label = str(species_C1)+' initial',linewidth=2)

plt.plot(x_array, 1000*dfC1.iloc[len(dfC1)-1], 'b',label = str(species_C1)+' final',linewidth=2)

if (c_C2_0 !=0) : plt.plot(x_array, 1000*dfC2.iloc[0], 'g:',label = str(species_C2)+' initial',linewidth=2)

if (c_C2_0 !=0) : plt.plot(x_array, 1000*dfC2.iloc[len(dfC2)-1], 'g',label = str(species_C2)+'final',linewidth=2)

plt.plot(x_array, 1000*dfA1.iloc[0], 'r:',label = str(species_S)+' initial',linewidth=1.5)

plt.plot(x_array, 1000*dfA1.iloc[len(dfA1)-1], 'r',label = str(species_S)+'final',linewidth=2)

#plt.plot(x_array, 1000*(z_A*dfA.iloc[len(dfA)-1]+z_B*dfB.iloc[len(dfB)-1]+z_C*dfC1.iloc[len(dfC1)-1]),'k',label = 'Amount charge')

plt.plot(x_array, 1000*(z_C1*dfC1.iloc[len(dfC1)-1]+z_C2*dfC2.iloc[len(dfC2)-1]-z_A1*dfA1.iloc[len(dfA1)-1]),'k:',label = 'c⁺ - c⁻')

plt.xlabel("distance ('+str(x_unit)+')'")

plt.ylabel("concentration (M)")

plt.legend()

plt.savefig(savepath+'//initial-final.png', bbox_inches = "tight", dpi = 200)

plt.show()

print('time evolution concentrations')

for i in time_series:

plt.plot(x_array, 1000*dfC1.iloc[int(i*(len(dfC1)-1))], label = str(int(t_total/t_res*i))+" "+t_unit,linewidth=2)

#plt.text(min(x_array) + 0.6*(max(x_array)-min(x_array)), min(1000*dfC1.iloc[int(1*(len(dfC1)-1))]), 'current = '+str(current)+' mA/cm²', fontdict=None)

#plt.title('current = '+str(current*1000)+' mA/cm²')

plt.xlabel('distance ('+str(x_unit)+')')

plt.ylabel("concentration (M)")

plt.legend(prop={'size': 14})

plt.savefig(savepath+'//c_C-time-evolution.png', bbox_inches = "tight", dpi = 200)

plt.ylim(0)

#plt.text(0.5*x_array.max(), 0.005, electrolyte,fontsize=15)

plt.show()

plt.plot(time_array, 1000*dfC1[list(dfC1)[10]], 'b:',label = str(species_C1)+' initial',linewidth=2)

plt.xlabel("c in 10th element (M)")

plt.ylabel("time (s)")

plt.show()

return current

# =============================================================================

# Trigger the function

# =============================================================================

# =============================================================================

# Experimental evaluation

# =============================================================================

limiting_current = True

current_profile = False

path_j_profile = ""

start_current = 0.015 # A/cm²

distance = 0.05 # cm

c_C1_0 = 0.1 # mol/l

time = 300

x_steps = 50

salt_1 = "CuSO4"

salt_2 = "Na2SO4"

# correction factors due to temperature variation from literature data

E.cf_CuSO4_mc = 0.91

E.cf_CuSO4_D = 0.88

E.cf_Na2SO4_D = 0.88

E.cf_Na2SO4_mc = 1

#path_j_profile = "current_profile.csv"

for interaction in ["none","individual","combined","combined_20"]:

saveroot = os.getcwd() + "/"+interaction

if os.path.exists(saveroot) == False: os.mkdir(saveroot)

concentration_var = [0,0.01,0.02,0.05,0.1,0.2,0.5,1]

current = 0.02

for i in range(0,len(concentration_var)):

c_C2_0 = concentration_var[i]

savepath = saveroot + "/Na_var"+str(i)

if os.path.exists(savepath) == False: os.mkdir(savepath)

model(savepath, distance, x_steps, salt_1, c_C1_0, salt_2, c_C2_0, time, path_j_profile)

# =============================================================================

# Parameter variation

# =============================================================================

salt_1 = "active_ions"

salt_2 = "supporting_ions"

c_C1_0 = 0.1

E.mc0["active_ions"] = 100

E.D0["active_ions"] = 1e-5

E.t0["active_ions"] = 0.5

E.mc0["supporting_ions"] = E.mc0["active_ions"]

E.D0["supporting_ions"] = E.D0["active_ions"]

E.t0["supporting_ions"] = E.t0["active_ions"]

interaction = "none"

current_profile = False

limiting_current = True

path_j_profile = ""

x_steps = 50

current_init = 0.02

start_current = current_init

distance = 0.1

time = 400

variation_factor = [0.001,0.003,0.01,0.03,0.1,0.3,1,3,10,30,100,300,1000]

# Reference

c_C2_0 = 0

savepath = "without_supporting"

if os.path.exists(savepath) == False: os.mkdir(savepath)

model(savepath, distance, x_steps, salt_1, c_C1_0, salt_2, c_C2_0, time, path_j_profile)

# Variation of concentration

for i in range(0,len(variation_factor)-1):

start_current = current_init

saveroot = os.getcwd() + "/var_c"

if os.path.exists(saveroot) == False: os.mkdir(saveroot)

savepath = saveroot + "/var"+str(i)

if os.path.exists(savepath) == False: os.mkdir(savepath)

c_C2_0 = c_C1_0*variation_factor[i]

model(savepath, distance, x_steps, salt_1, c_C1_0, salt_2, c_C2_0, time, path_j_profile)

c_C2_0 = c_C1_0

# Variation of molar conductivity

for i in range(0,len(variation_factor)):

saveroot = os.getcwd() + "/var_mc"

if os.path.exists(saveroot) == False: os.mkdir(saveroot)

savepath = saveroot + "/var"+str(i)

if os.path.exists(savepath) == False: os.mkdir(savepath)

E.mc0["supporting_ions"] = E.mc0["active_ions"]*variation_factor[i]

model(savepath, distance, x_steps, salt_1, c_C1_0, salt_2, c_C2_0, time, path_j_profile)

E.mc0["supporting_ions"] = E.mc0["active_ions"]

c_C2_0 = c_C1_0

# Variation of diffusivity

time_array = [2000, 2000, 1000, 1000, 1000, 800, 400, 300, 100, 50, 20, 15, 10]

for i in range(0,len(variation_factor)):

saveroot = os.getcwd() + "/var_D"

if os.path.exists(saveroot) == False: os.mkdir(saveroot)

savepath = saveroot + "/var"+str(i)

if os.path.exists(savepath) == False: os.mkdir(savepath)

E.D0["supporting_ions"] = E.D0["active_ions"]*variation_factor[i]

time = time_array[i]

model(savepath, distance, x_steps, salt_1, c_C1_0, salt_2, c_C2_0, time, path_j_profile)

E.D0["supporting_ions"] = E.D0["active_ions"]
